# CANNABIS USE AMONG OLDER ADULT CANCER PATIENTS: PATTERNS OF USE AND PERCEIVED SYMPTOM MANAGEMENT

**DOI:** 10.1093/geroni/igad104.0565

**Published:** 2023-12-21

**Authors:** Margaret Fahey

**Affiliations:** Medical University of South Carolina, Charleston, South Carolina, United States

## Abstract

Prevalence of cannabis use among U.S. older adults (>65 years) is increasing and is common among cancer survivors of all ages. This National Cancer Institute-funded supplement supported administration of a cross-sectional survey in a state with illegal access to cannabis. This study descriptively examined weighted estimates of cannabis rates (lifetime, post-diagnosis, current) among older patients (>65 years) (N=524; 51% of full sample) with a cancer diagnosis (< two years). Of those endorsing post-diagnosis use, weighted estimates examined patterns of use (timing relative to cancer treatment, reasons for use, primary mode of use) and self-reported impact of cannabis on symptoms (since diagnosis; 5-point Likert scale for 9 symptoms). Almost half (46.0%) of older patients used cannabis in their lifetime, with 18.2% using post diagnosis and 9.8% currently using. Of patients who used cannabis following diagnosis (N=107), approximately half used during cancer treatment (7.0% had not yet started) and most common mode of use was smoking (e.g., joint) followed by edible use. Most common reasons were for pain (44.3%), sleep (43.4%) and recreationally (38.1%). Most common symptoms patients endorsed improved quite a bit, included pain (27.7%), difficulty sleeping (27.3%), and stress, anxiety, depression (23.5%). Overall, few believed cannabis worsened symptoms, with most indicating either improvement or no change. Findings indicate older oncology patients commonly use cannabis to alleviate medical or health symptoms. Oncology care teams require evidence-based recommendations and guidance to inform conversations and shared decision making about cannabis with their older patients, even within a state without a legal cannabis marketplace.

